# GazeBaseVR, a large-scale, longitudinal, binocular eye-tracking dataset collected in virtual reality

**DOI:** 10.1038/s41597-023-02075-5

**Published:** 2023-03-30

**Authors:** Dillon Lohr, Samantha Aziz, Lee Friedman, Oleg V. Komogortsev

**Affiliations:** Texas State University, Department of Computer Science, San Marcos, TX 78666 USA

**Keywords:** Computer science, Electrical and electronic engineering

## Abstract

We present GazeBaseVR, a large-scale, longitudinal, binocular eye-tracking (ET) dataset collected at 250 Hz with an ET-enabled virtual-reality (VR) headset. GazeBaseVR comprises 5,020 binocular recordings from a diverse population of 407 college-aged participants. Participants were recorded up to six times each over a 26-month period, each time performing a series of five different ET tasks: (1) a vergence task, (2) a horizontal smooth pursuit task, (3) a video-viewing task, (4) a self-paced reading task, and (5) a random oblique saccade task. Many of these participants have also been recorded for two previously published datasets with different ET devices, and 11 participants were recorded before and after COVID-19 infection and recovery. GazeBaseVR is suitable for a wide range of research on ET data in VR devices, especially eye movement biometrics due to its large population and longitudinal nature. In addition to ET data, additional participant details are provided to enable further research on topics such as fairness.

## Background & Summary

Eye-tracking (ET) sensors are becoming increasingly prevalent in modern virtual- and augmented-reality (VR/AR) devices such as the Vive Pro Eye^1^, HoloLens 2^2^, and Magic Leap 2^3^. The presence of these ET sensors is motivated in large part to enable foveated rendering techniques^4^ which offer a significant reduction in overall power consumption without a noticeable impact on visual quality. Such power savings could lead to higher resolution displays in tethered devices or a longer battery life in untethered devices. In addition to foveated rendering, ET also enables a multitude of applications including (continuous) user authentication^5,6^, health monitoring^7^, novel display technologies^8^, usability assessment^9^, direct gaze interaction^10^, and more.

Research on these applications is heavily dependent on the availability of large-scale datasets. Eye movement biometrics (EMB)^11^, especially, requires large, longitudinal datasets with hundreds of unique identities and varied eye movement behaviors to train state-of-the-art deep learning models^5,12^. One of the most suitable datasets for EMB is GazeBase^13^, a dataset of high-quality monocular (left eye only) ET signals recorded at 1000 Hz over a 37-month period from a population of 322 college-aged participants. However, at the time of writing, there is no similar, large-scale, longitudinal dataset collected with a modern VR/AR device, making it difficult to train an EMB model tailored to such devices.

**Fig. 1:**
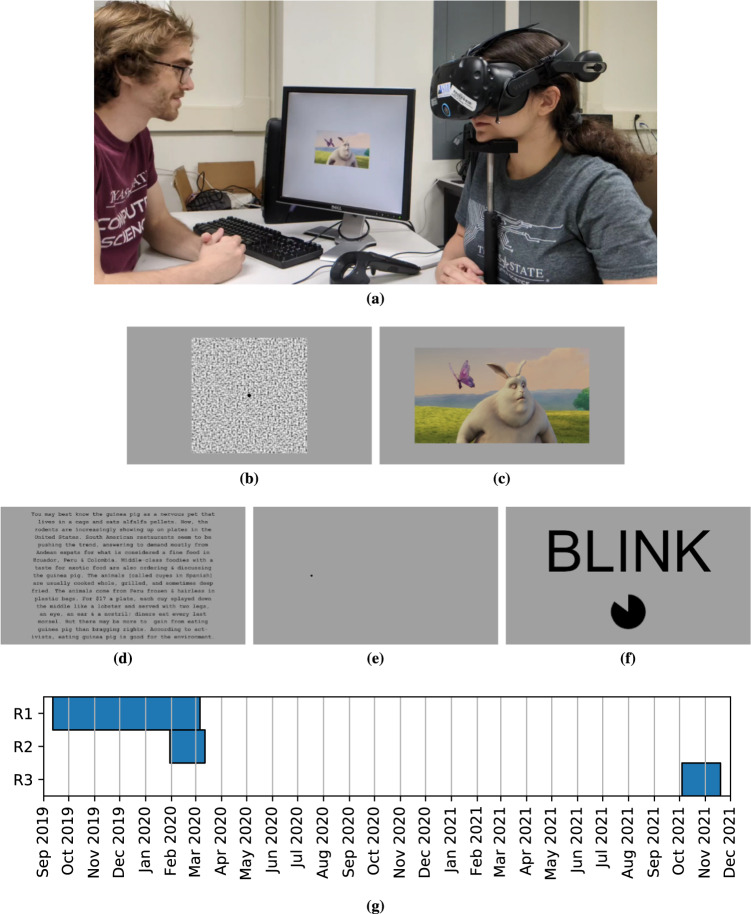
Summary of the GazeBaseVR dataset collection. (**a**) An illustration of the experimental setup. The depicted individuals gave permission to be shown here. (**b**) The stimulus used for the vergence task. (**c**) A frame from one of the video clips used for the video task. (**d**) One of the text excerpts used for the reading task. (**e**) The dot stimulus used for the smooth pursuit and random saccade tasks. (**f**) The text and timer displayed during the “blink period” prior to each task. (**g**) The time periods during which each recording round took place.

The present work introduces GazeBaseVR, a GazeBase-inspired dataset collected with an ET-enabled VR headset. GazeBaseVR contains binocular ET signals recorded at 250 Hz over a 26-month period from a diverse population of 407 college-aged participants. A summary of the GazeBaseVR dataset collection is presented in Fig. 1. One particularly noteworthy component of GazeBaseVR is a task that elicits vergence eye movements which are underrepresented in public ET datasets. We also note that a sampling rate around 250 Hz, which GazeBaseVR has, is commonly considered to be necessary to accurately capture saccade characteristics such as peak velocity and duration^14^. A subset of this dataset was described and used in a brief prior study^15^, but this is the first release of the full dataset.

Further, some of the participants from GazeBaseVR were also later recorded for two other public datasets: SynchronEyes^16^ and the HoloLens 2 ET dataset by Aziz and Komogortsev^17^. This overlap in populations may enable research on the generalizability of EMB models across several different ET devices, among other potential applications. While many prior studies have also recorded a set of participants with multiple ET devices^18–21^, existing datasets tend to either not be publicly available, not contain enough unique identities for a robust analysis, or not contain sufficiently varied eye movement behaviors for applications such as EMB.

When considering population size, task diversity, and temporal scale, GazeBaseVR is–to the best of our knowledge–the largest publicly available dataset of ET signals collected with a VR (or AR) device. A comparison of GazeBaseVR to a selection of similar, publicly available datasets is given in Table 3. Existing ET datasets are predominantly recorded outside of VR/AR devices, making it difficult to perform studies on data from VR/AR devices. Aside from GazeBase, existing datasets tend to have a limited longitudinal aspect; but some areas of research such as EMB benefit from the ability to study long-term patterns in ET signals. Compared to GazeBase, GazeBaseVR offers a greater number of participants (407 vs 322), binocular recordings rather than monocular ones, and the addition of vergence and smooth pursuit tasks to elicit novel types of eye movement. Though, GazeBase spans a longer period of time than GazeBaseVR (37 months vs 26 months) and contains a greater number of recordings per participant.

## Methods

### Participants

**Fig. 2:**
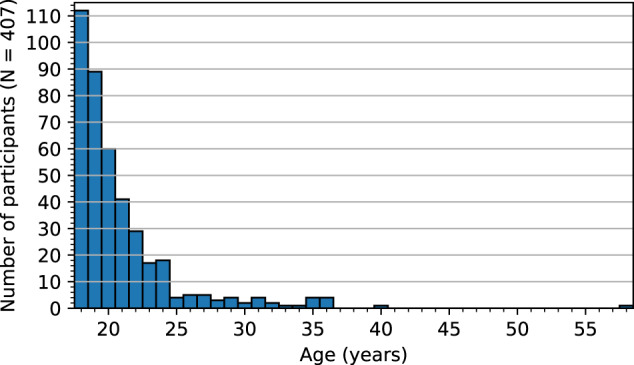
Distribution of participants’ ages at enrollment time (i.e., the first session of the first recording round).

A total of 465 individuals originally participated in the study, but 58 participants were excluded for various reasons (e.g., could not be tracked/calibrated, experienced motion sickness, could not finish within the allotted time of 1 hour, excessive (over 50%) data loss in one or more recordings). At the time of Round 1, 188 participants self-identified as male, 216 self-identified as female, and 3 self-identified as neither male nor female. See Table 1 for race/ethnicity statistics and Fig. 2 for the distribution of participants’ ages at enrollment time. A total of 3 recording rounds took place over a period of 26 months (see Table 2 for date ranges and population sizes), with each round comprising 2 recording sessions separated by approximately 30 minutes. Round 2 began alongside a continuation of Round 1 in the beginning of the Spring 2020 semester, but recordings were prematurely halted due to health concerns at the start of the global COVID-19 pandemic. Recordings later resumed with Round 3, throughout which the laboratory personnel wore face masks at all times and participants wore face masks except when actively being recorded to reduce health risks, and extra care was taken to disinfect all the equipment after each set of recordings. During Round 3, 11 participants were recorded after recovering from a COVID-19 infection, which may have affected their eye movements in some way^22^; but this is too small of a sample size to perform any meaningful analysis on the effects of COVID-19 on ET data.

**Table 1:** Self-reported race/ethnicity of the participants at the time of Round 1.

Race/ethnicity	Number of participants
American Indian or Alaska Native	0
Asian	11
Black or African American	41
Hispanic or Latino	148
Native Hawaiian or Other Pacific Islander	1
White	140
Mixed	62
Prefer not to answer	4

Participants who self-identified as two or more options are classified as “mixed.”

**Table 2:** The number of participants and dates of the first and last recordings for each recording round.

Recording round	Number of participants	Start date	End date
R1	407	2019-09-12	2020-03-06
R2	35	2020-01-30	2020-03-12
R3	60	2021-10-04	2021-11-19

Dates are given in YYYY-MM-DD format. Although Round 2 was collected concurrently with Round 1, the minimum separation between a participant being recorded for R1 and R2 was 84 days.

There are fewer participants in Round 2 than the other rounds because it was prematurely halted due to COVID-19, so participants for Round 3 were recruited from the Round 1 population. The participants in Rounds 2 and 3 are both subsets of the population from Round 1, but a participant in Round 2 may not be present in Round 3 and vice versa. To be precise, 322 participants were present only for Round 1, 75 participants were present for Round 1 and one other round (25 for Round 2 and 50 for Round 3), and 10 participants were present for all three rounds. It may be worth briefly expanding upon the reason behind this distribution of participants across recording rounds. At the onset of data collection, we had originally planned to follow a similar scheme as GazeBase wherein subsequent recording rounds would recruit exclusively from the preceding round’s participants. However, Round 2 was prematurely halted due to COVID-19, resulting in an unexpectedly small population for that round. When recordings later resumed once it was deemed safe to do so, too much time had passed for a continuation of Round 2 to be sensible. So for Round 3, rather than limit ourselves to the relatively small population of Round 2, we decided to maximize the population of Round 3 by recruiting from the full Round 1 population.

Participants were recruited from the undergraduate student population at Texas State University in San Marcos, TX, USA. All participants were screened to ensure they had no history of epilepsy or seizures, and they all provided informed, written consent to participate in the study and to have their anonymized data shared with the broader research community following a protocol approved by the Institutional Review Board at Texas State University. The protocol was later modified for Round 3 to abide by Texas State University’s COVID-19 health and safety guidelines, and this amended protocol was also approved by the Institutional Review Board at Texas State University. Participants were compensated with extra course credit for participation in Round 1, $20 in Round 2, and $40 in Round 3. The increase in monetary compensation for participation in Round 3 was done to decrease attrition rates and was approved as part of the modifications made to the Round 3 protocol.

### Data acquisition overview

ET data are recorded with SensoMotoric Instrument’s (SMI’s) tethered ET VR head-mounted display based on the HTC Vive (hereon called the ET-HMD). The ET-HMD tracks both eyes at a nominal sampling rate of 250 Hz with a manufacturer-reported typical spatial accuracy of 0.2 degrees of visual angle (dva) and–according to correspondence we had with SMI support prior to Apple’s acquisition of SMI–a manufacturer-reported spatial precision of “about 0.02” dva. The experiments were designed in Unity 2018.3.11f1 using the C# programming language.

When deciding which ET-enabled device to use for data collection, we could have chosen either VR or AR to target the kinds of devices that would greatly benefit from the inclusion of ET sensors to enable foveated rendering. The primary reason we chose to use a VR device over an AR device was due to the availability of the ET-HMD and the fact that, at the time that data collection began, it was equipped with the highest quality ET sensors available in a VR or AR device. More generally, VR enables more immersive entertainment experiences than AR and makes it easier to develop ET tasks without concern for visual distractions in the surrounding environment. There may be differences in the level of ET signal quality accessible in AR devices compared to VR devices, so our proposed dataset may not be ideal for studies specifically interested in ET signals captured with an AR device.

To facilitate a better and more comfortable headset fit, the stock head strap was replaced by the HTC Vive Deluxe Audio Strap, but no audio was ever played during the experiments. Additionally, the stock 14 mm foam face cushion was replaced with a 6 mm polyurethane leather face cushion to increase the field of view within the headset and make it easier to clean.

The view in the headset is fixed during each task so that, regardless of any head movement, each stimulus maintains the correct position relative to the headset. A participant puts on the headset, adjusts its fit for comfort and image clarity with assistance from the recording administrator as needed, and rests his/her head on a chin rest to minimize head movements. Although the view in the headset is fixed, it is still desirable to minimize head movements with a chin rest to reduce headset slippage, to reduce the risk of discomfort caused by the fixed view, and to reduce unintended eye movement behavior caused by the vestibulo-ocular reflex.

Data collection was performed by a combination of undergraduate students, graduate students, and post-doctoral researchers who were trained and followed a standardized procedure for ensuring proper headset fit and maximizing the signal quality of each recording. A gaze cursor was displayed on the monitor (but not within the headset) at all times to allow the recording administrator to identify problems such as excessive noise or extreme inaccuracy, and each recording administrator was trained to identify and resolve these issues involving means such as readjusting the headset (which was made easier by the use of the deluxe audio strap), cleaning the lenses, or recalibrating. Together with the use of a chin rest to minimize head movements, these measures would have mitigated the potential for headset slippage. However, beyond these measures, no special care was taken to completely eliminate headset slippage.

### Calibration and validation

Participants perform a manufacturer-provided calibration procedure at scheduled intervals prior to the vergence, reading, and random saccade tasks, or whenever the headset is removed for any reason. The calibration procedure involves following a moving dot in a standard 5-point grid pattern. Calibration is performed up to 3 times until a spatial accuracy below 1 dva is achieved, moving on after the third attempt regardless of spatial accuracy. Spatial accuracy is assessed after each calibration attempt with a short, custom validation procedure consisting of a 13-point grid spanning ±15 dva horizontally and ±10 dva vertically at a depth of 1 meter.

In an effort to reduce fatigue, calibration is not performed prior to every task. This is justified by the use of a head-mounted display and a chin rest, as significant headset slippage is unlikely and high tracking accuracy can be maintained for longer periods than may be expected for non-wearable ET devices.

### Task battery overview

An ordered series of 5 eye-tracking tasks are performed during each recording session. The tasks are designed to elicit a variety of commonly studied types of eye movement. Some tasks are intended to make it easier to isolate a particular type of eye movement (e.g., convergent/divergent movements in the vergence task, smooth pursuit movements in the smooth pursuit task, and saccadic/fixation movements in the random saccade task), while others are intended to elicit a complex mixture of different types of eye movement (e.g., saccadic, smooth pursuit, and fixation movements in the video viewing task). Each task is described in the following subsections in the order they occur within each session. The task abbreviations included in the subsection titles are part of the file naming convention for GazeBaseVR.

Each task is preceded by a 3-second-long “blink period” during which participants are instructed to blink as needed in an effort to reduce the amount they would need to blink during the task itself. Any eye-tracking data recorded during these blink periods is discarded. During this period, the text “BLINK” appears in large, black font over a light-gray background. Below the text is a black radial wipe timer that participants can use to gauge how much time remains until the task begins. Participants are instructed to try to minimize their blinks during each task and, if they need to blink, to try to blink only during periods when the visual target is stationary. See Fig. 1f for a visualization of the blink period.

All tasks including the blink period have the same light-gray background color (hex: #A0A0A0, RGB: (160, 160, 160)). The background color used in the manufacturer-provided calibration procedure is a slightly darker gray (hex: #7F7F7F, RGB: (127, 127, 127)).

#### Task 1: Vergence task (VRG)

This task is modeled after a study on the dynamics of vergence eye movements by Tyler *et al*.^23^ During the task, a large (30 × 30 dva), square plane textured with random, gray-scale noise is displayed in the center of the user’s field of view. At the center of the plane is a small (1 dva diameter), black sphere on which participants are instructed to focus throughout the task. The stimulus alternates between depths of approximately 0.4433 and 0.3543 meters, eliciting ideal vergence (left minus right) of 8 and 10 dva, respectively, assuming an interocular distance of 62 mm. The stimulus scales in size with changes in depth to maintain a constant apparent size so that vergence eye movements are driven by image disparity alone. Periods between depth changes are uniformly random between 2 and 3 seconds. The task has a duration between 48 and 72 seconds and elicits a total of 12 convergent (toward the nasal bridge) and 12 divergent (away from the nasal bridge) eye movements. See Fig. 1b for a visualization of the stimulus for this task.

#### Task 2: Smooth pursuit task (PUR)

During this task, a small, black sphere (0.5 dva diameter at 1 meter depth) glides smoothly between the left and right edges of the viewing region (±15 dva) to elicit horizontal smooth pursuit eye movements. The stimulus begins at the center of the screen and, after a delay of 1.5 seconds, smoothly moves to the left edge of the viewing region at a constant speed of 5 dva/s. After a random delay between 1 and 1.5 seconds, it smoothly moves from the left edge to the right edge, pauses for another random delay when it reaches the right edge, and then smoothly moves back to the left edge. We refer to this complete left-to-right-to-left movement as a “trap,” since when plotting the horizontal position of the stimulus versus time its shape resembles a trapezoid. The stimulus performs as many complete traps as necessary to satisfy at least 30 seconds of movement, not including the random pauses at the left and right edges nor the time it takes to move to and from the center of the screen. It then returns to the center of the viewing region, pauses for 1.5 seconds, and repeats the full movement pattern at a higher speed.

A total of 3 different speeds are employed during this task in a fixed order: 5 dva/s, 10 dva/s, and 20 dva/s. At 5 dva/s, the stimulus performs 3 traps totaling 36 seconds of movement, plus an additional 6 seconds moving to and from the center of the screen. At 10 dva/s, the stimulus performs 5 traps totaling 30 seconds of movement, plus an additional 3 seconds moving to and from the center of the screen. At 20 dva/s, the stimulus performs 10 traps totaling 30 seconds of movement, plus an additional 1.5 seconds moving to and from the center of the screen. Together with the pauses at the edges and center, the task has a total duration between 151.5 and 171 seconds. See Fig. 1e for a visualization of the stimulus for this task.

#### Task 3: Video viewing task (VID)

During this task, a video (1280 × 720 resolution, 30 frames per second) is displayed on a large, rectangular plane (36 × 21 dva at 1 meter depth) in the center of the user’s field of view over a light-gray background. Participants are instructed to view the video as they normally would. The video is a clip from the 3D animated short film, Big Buck Bunny^24^, with different clips being used for each session and the same two clips being used for all recording rounds. The first session uses the clip between timestamps 01:50–02:28 (38 seconds duration) and the second session uses the clip between timestamps 05:45–06:23 (38 seconds duration). Each clip involves periods where one or more objects of interest are moving or stationary, eliciting a variety of eye movement behaviors. See Fig. 1c for a visualization of the stimulus for this task. The clips used for both sessions are provided with the supplementary code on figshare^25^. While it is perhaps more common in VR to view 360° video, we opted to display the video on a plane to more closely match the video presentation format of GazeBase.

#### Task 4: Reading task (TEX)

During this task, an excerpt (roughly 820 characters) of an article from National Geographic is displayed within a 51.2 × 37.6 dva viewing region at a depth of 0.6 meters in the center of the user’s field of view. The chosen text contains easily digestible, non-fiction prose. A fixed-width font is used such that each character has a width close to 1 dva (varying with eccentricity). The font is black and is displayed over a light-gray background.

Participants hold an HTC Vive controller during the task and are instructed to press the rear trigger button to indicate that they have finish reading the text. Afterward, a multiple-choice reading comprehension question is displayed in the headset and participants must select one of the four answer choices using the controller to complete the task. Participants are informed beforehand that there will be a reading comprehension question. The question is not intended to be difficult, and the correctness of the selected answer is irrelevant; the purpose of this question is to encourage participants to read the text closely and not merely skim through it. The selected answer choice and any eye-tracking data recorded while answering the question are discarded.

The duration of the task depends on how quickly a participant reads through the text, ranging from 22.2 to 141.4 seconds (median 51.6, IQR 17.9). A total of 4 unique text excerpts were used throughout data collection: one for session 1 of Round 1, one for session 2 of Round 1, one for session 1 of Rounds 2 and 3, and one for session 2 of Rounds 2 and 3. See Fig. 1d for a visualization of the stimulus for this task.

#### Task 5: Random saccade task (RAN)

During this task, a small, black sphere (0.5 dva diameter at 1 meter depth) begins at the center of the user’s field of view and jumps to uniformly random positions on the screen within ±15 dva horizontally and ±10 dva vertically. There is a uniformly random delay between 1 and 1.5 seconds and a minimum distance of 3 dva separating consecutive jumps. Participants are instructed to focus on and follow the sphere with their eyes throughout the task. A total of 79 stimulus movements (80 fixation periods) occur throughout the task, resulting in a duration between 80 and 120 seconds. See Fig. 1e for a visualization of the stimulus for this task.

## Data Records

GazeBaseVR is available for download on figshare^26^ under a Creative Commons Attribution 4.0 International (CC-BY 4.0) license. In addition to the ET data, a file named participant_details.xlsx is included with many self-reported details for each participant, including but not limited to age, gender, race/ethnicity, eye dominance, sleepiness on the Stanford Sleepiness Scale^27^, drug and alcohol use, and physical and mental health. All recordings and the additional participant details file have been anonymized in accordance with the informed consent provided by all participants.

The ET API provided by SMI produces 3-dimensional unit vectors representing the gaze direction of each eye and timestamps with nanosecond precision. The API uses the same (left-handed) coordinate system as Unity such that positive X points right, positive Y points up, and positive Z points into the screen (i.e., the eyes look toward positive Z). A direction vector **v** = [*x, y, z*] is converted to the horizontal (*θ*_*H*_) and vertical (*θ*_*V*_) components of the rotation of the eye globe in terms of dva using the equations

1 $${\theta }_{H}=\frac{180}{\pi }{\rm{atan}}2\left(x,\sqrt{{y}^{2}+{z}^{2}}\right)$$

2 $${\theta }_{V}=\frac{180}{\pi }{\rm{atan}}2\left(y,z\right),$$

where atan2 is the four-quadrant inverse tangent. These equations are analogous to the formulas for converting from Cartesian coordinates to spherical coordinates, taking into account the coordinate system used in the ET API provided by SMI. At each time step, a direction vector is provided for the left eye, right eye, and cyclopean eye (called the “camera raycast” in the ET API). Each eye’s direction vector may separately be reported as NaN in cases where the gaze could not be estimated including, but not necessarily limited to, during blinks. For tasks with a dot stimulus, the same equations are used to convert the position of the stimulus in world coordinates to dva relative to the cyclopean eye. The timestamps are scaled from nanoseconds to milliseconds and transformed to always start from 0. These units of measure–timestamps reported as milliseconds since the beginning of the recording and gaze positions reported as dva–are similar to those used in GazeBase. In addition, GazeBaseVR also includes the 3D position in meters (relative to the camera origin) of each eye ball as reported by the ET API.

There may be some latency involved with querying the position of the GameObject associated with the stimulus from within the ET API’s callback function. There may also be some latency between the eye image being captured by the ET sensors and the resulting gaze data being made available via the callback function. But we do not have detailed information regarding these latencies.

**Fig. 3:**
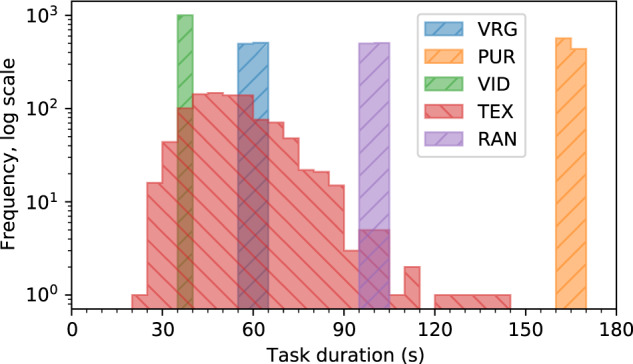
Distributions of the duration of each task across all recording rounds, sessions, and participants. A bin width of 5 seconds is used for each histogram.

Data files are provided in CSV format inside a subdirectory named “data” following a naming convention similar to that of GazeBase: S_rxxx_Sy_z_www.csv. Table 4 describes the components of the file naming convention, and Table 5 describes the contents of each CSV file. The distributions of recording duration grouped by task are presented in Fig. 3.

**Table 3:** Comparison of GazeBaseVR to a selection of similar, publicly available datasets collected both in and out of VR/AR.

Dataset	Rate (Hz)	Eyes*	Tracker type	N	No. sessions	Session gap	Task diversity
GazeBaseVR (ours)	250	L/R/C	VR headset	407	2–6	30 min.–2 yr.	Vergence; smooth pursuit; video; reading; 2D saccades
GazeBase^13^	1000	L	Tower mount	322	2–18	30 min.–3 yr.	Video; reading; 1D saccades; 2D saccades; fixation; interactive game
JuDo1000^33^	1000	L/R	Tripod mount	150	4	1 wk.–4 wk.	2D saccades
DGaze^34^	100	C	VR headset	43	2	Same day	Dynamic 360° scenes
SynchronEyes^16^	500/1000	Varies	Wearable and tower mount	20	2	Same day	Video; reading; 1D saccades; 2D saccades; fixation
Aziz and Komogortsev^17^	30	L/R	AR headset	33	1	—	2D saccades

“N” represents the total number of unique participants. “No. sessions” represents how many times a participant completes a full set of tasks. “Session gap” represents the approximate temporal separation between any two recording sessions.

*L = left eye, R = right eye, C = cyclopean eye.

**Table 4:** Description of file naming convention: S_rxxx_Sy_z_www.

Filename component	Description	Possible values
r	Recording round	1–3
xxx	Participant identifier	001–465
y	Recording session	1–2
z	Task number	1–5
www	Task code	VRG, PUR, VID, TEX, RAN

**Table 5:** Description of data format. Note that dva is reported in degrees rather than radians, with decimal values indicating fractions of an angle rather than minutes/seconds of arc.

Column header	Unit of measure	Description
n	ms	timestamp of the recorded gaze sample since the beginning of the recording
x	dva	*θ*_*H*_ for the cyclopean eye
y	dva	*θ*_*V*_ for the cyclopean eye
lx	dva	*θ*_*H*_ for the left eye
ly	dva	*θ*_*V*_ for the left eye
rx	dva	*θ*_*H*_ for the right eye
ry	dva	*θ*_*V*_ for the right eye
xT*	dva	*θ*_*H*_ for the stimulus, relative to the cyclopean eye
yT*	dva	*θ*_*V*_ for the stimulus, relative to the cyclopean eye
zT	m	depth of the stimulus
clx	m	X position of the center of the left eye ball, relative to the camera origin
cly	m	Y position of the center of the left eye ball, relative to the camera origin
clz	m	Z position of the center of the left eye ball, relative to the camera origin
crx	m	X position of the center of the right eye ball, relative to the camera origin
cry	m	Y position of the center of the right eye ball, relative to the camera origin
crz	m	Z position of the center of the right eye ball, relative to the camera origin

*Only provided for tasks with a dot stimulus (VRG, PUR, and RAN). NaN for all other tasks.

## Technical Validation

In terms of ET signal quality, the ET-HMD was one of the best ET-enabled VR headsets when it was released, boasting an impressive 250 Hz sampling rate and a manufacturer-reported typical spatial accuracy of just 0.2 dva. Competition at the time included devices such as the Vive Pro Eye^1^ with a sampling rate of 120 Hz and a manufacturer-reported spatial accuracy of 0.5–1.1 dva, the FOVE 0^28^ with a sampling rate of 120 Hz and a manufacturer-reported spatial accuracy of less than 1 dva, and the Varjo VR-1^29^ with a sampling rate of 100 Hz and a manufacturer-reported spatial accuracy of less than 1 dva.

Unlike other ET devices such as the EyeLink 1000 which provide spatial accuracy measurements during a manufacturer-provided validation procedure, we are not aware of a built-in method to quantitatively measure the spatial accuracy and spatial precision of the ET-HMD, at least when using the ET API within Unity. Therefore, signal quality must be measured in a user-specified manner. A highly accurate measure of signal quality would require careful classification of every stable fixation in every recording, which is a deceptively difficult task and beyond the scope of this work. We therefore choose to measure the spatial accuracy and spatial precision of the dataset in the following manner, based on the broadly applicable methodology from Lohr *et al*.^30^.

First, we minimize saccade latency in the gaze position signal. This is accomplished by temporally shifting the gaze position signal backward one time step at a time (up to a limit of 200 time steps or approximately 800 ms) and measuring the mean Euclidean distance between the shifted gaze position signal and the unshifted stimulus position signal. The temporal shift resulting in the lowest mean Euclidean distance is applied to the gaze position signal, effectively minimizing saccade latency. Second, we partition the signal into different “fixation periods,” using the time steps when the stimulus changes position to separate adjacent fixation periods. Third, we ignore the first 100 time steps (approximately 400 ms) of each resulting fixation period, because this is generally a period of instability as the eye finishes saccadic movement and settles into stable fixation at the stimulus’ current position. Finally, we employ the next 125 time steps (approximately 500 ms) of each fixation period for signal quality evaluation. We utilize the cyclopean gaze signal from the Session 1 RAN task, and we consider only the first 20 fixation periods of each recording to reduce the influence of fatigue effects on our measurements.

To measure spatial accuracy, for each fixation period, we compute the Euclidean distance between each gaze position sample and the stimulus position during that fixation period. We compute the mean of these distances within each fixation period and then the mean across fixation periods within a recording.

To measure spatial precision, for each fixation period, we compute the Euclidean distance between each consecutive gaze position sample. We compute the root mean square (RMS) of these inter-sample distances within each fixation period and then the mean across fixation periods within a recording. RMS is computed using the equation

3 $${\rm{RMS}}=\sqrt{\frac{1}{N}\mathop{\sum }\limits_{i=1}^{N}{x}_{i}^{2}},$$

where *x*_*i*_ is the *i*-th inter-sample distance. This method of measuring spatial precision is one of the two most common methods, the other being to compute the standard deviation of gaze position samples^31^.

The resulting signal quality measurements are likely pessimistic estimates of the true signal quality, considering that saccadic movements and artifacts (e.g., due to blinks) may be present in the samples used for the computations. Based on these rough measurements, all 3 rounds have a median spatial accuracy of around 0.9–1.0 dva and a median spatial precision of around 0.03–0.04 dva. Although this is significantly worse than the manufacturer-reported spatial accuracy of 0.2 dva, it is well known that manufacturer-reported signal quality measurements are often not achievable in practice^32^ .

**Fig. 4:**
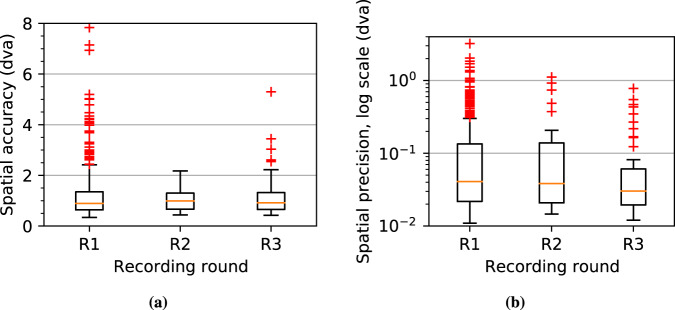
Rough measurements of (**a**) spatial accuracy and (**b**) spatial precision for each participant and each recording round, using the broadly applicable methodology of Lohr *et al*.^30^ to determine which samples to include in the measurements. Both quantities are measured using the cyclopean gaze signal from the session 1 RAN task, averaged over the first 20 fixation periods. Spatial precision is measured using the root mean square (RMS) of sample-to-sample distances, which is one of the two most common methods (the other being the standard deviation of gaze position samples)^31^.

## Data Availability

During data collection, raw CSV files were generated from the data stream accessed with SMI’s provided ET API within Unity. These raw CSV files were later converted to the format described in Table 5 using custom Python code. The code used to convert the raw files to the final format, along with the code used to generate Figs. 1g–4 and the data for Tables 1, 2, is available on figshare^25^. This code was developed using Python 3.7.11 with the following main packages: numpy 1.21.6, pandas 1.3.5, openpyxl 3.0.9, and matplotlib 3.2.2. This repository also contains other supplementary material including a manual for the ET-HMD, a pamphlet with manufacturer-provided technical specifications for the ET-HMD, and the video clips used for the VID task.
